# Assessment of minimally invasive caries excavation methods on caries-affected human dentin: A comparative analysis

**DOI:** 10.4317/jced.62599

**Published:** 2025-06-01

**Authors:** Ria Chawla, Preethesh Shetty, Raksha Bhat

**Affiliations:** 1 Nitte (Deemed to be University), AB Shetty Memorial Institute Of Dental Sciences(ABSMIDS), Department of Conservative Dentistry and Endodontics, Mangalore – 575018

## Abstract

**Background:**

Dental caries is a major global health issue, leading to tooth loss, infections, and even systemic complications. Modern dentistry favours minimally invasive caries removal, but its impact on the bond between composite and caries-affected dentin requires further study. The present in vitro analysis evaluated the microtensile bond strength (μTBS) of composite bonded to human dentin impacted by caries, after three caries removal methods: SmartPrep bur, chemo-mechanical removal, and air abrasion. The null hypothesis posited no significant difference in bond strength among the techniques.

**Material and Methods:**

Twenty-one extracted human molars with caries, but no pulpal involvement, were randomly allocated into three groups. Caries removal was performed using: 1) SmartPrep bur, 2) chemo-mechanical method using Papacarie® gel (Formula & Acao, Sao Paulo, Brazil) 3) air abrasion (AquaCare air-abrasion (Velopex, London, UK). Following caries removal, a two-step etch-and-rinse adhesive and composite restoration were placed. The test specimens from each group underwent microtensile bond strength testing utilizing a Universal Testing Machine, data were statistically analysed using one-way ANOVA and further examined with Tukey’s post hoc test (*p*<0.05).

**Results:**

Analysis revealed that SmartPrep bur demonstrated superior microtensile bond strength (33.31 ± 10.23 MPa), followed by chemo-mechanical removal (22.86 ± 11.61 MPa). Air abrasion yielded the lowest mean bond strength values (15.74 ± 2.99 MPa). The SmartPrep bur and air abrasion groups showed statistically significant differences (*p* = 0.012).

**Conclusions:**

This study demonstrated that the caries removal technique significantly influences the bond strength of composite to caries-affected dentin. The SmartPrep bur achieved superior microtensile bond strength, suggesting potential clinical benefits. These results emphasize the importance of technique selection for achieving optimal restorative outcomes.

** Key words:**Caries, Dentin, adhesion, tooth.

## Introduction

Dental caries represents a significant global public health concern, with implications ranging from tooth loss and localized infection to potentially fatal systemic complications (1). The foundational principles established by G.V. Black in the late 19th century, particularly the “extension for prevention” doctrine, dominated operative dentistry for over a century (2). However, contemporary understanding of cariology and the development of adhesive restorative materials have precipitated a paradigm shift toward tissue-preserving approaches, rendering traditional extensive cavity preparations increasingly obsolete (3).

Current evidence demonstrates that carious dentin exhibits distinct stratification: an external layer characterized by irreversible denaturation and extensive bacterial colonization, and an internal layer displaying reversible denaturation with preserved collagen architecture amenable to remineralization (4). In clinical scenarios, the residual caries-affected dentin frequently serves as the primary substrate for adhesive bonding procedures (5). Conventional rotary instrumentation generates significant mechanical and thermal stresses within the dentinal-pulpal complex, necessitating local anesthesia and potentially compromising pulpal health (6). Consequently, minimally invasive preparation techniques have emerged as evidence-based alternatives, prioritizing maximum conservation of sound tooth structure while maintaining biological and mechanical principles (7,8).

The efficacy of adhesive bonding is significantly influenced by the characteristics of the smear layer generated during cavity preparation. This microscopic debris layer, varying in morphology and thickness according to the preparation technique employed, can impede optimal adhesive infiltration unless adequately modified or removed (9,10). The present *in vitro* investigation aims to quantitatively assess and compare the microtensile bond strength values of composite resin bonded to caries-affected human dentin using three distinct caries removal techniques in conjunction with a resin-based adhesive system. The null hypothesis postulates no statistically significant differences in bond strength values among the three caries removal methodologies evaluated.

## Material and Methods

- Sample Collection

Twenty-one human permanent molars were carefully selected, each presenting carious lesions that extended at least halfway from the enamel-dentine junction to the pulp chamber. Prior to use, all specimens were evaluated for structural integrity and underwent disinfection protocols in strict adherence to the regulations set forth by the Occupational Safety and Health Administration (OSHA). Any specimens displaying structural compromise due to the extraction process or exhibiting extensive carious lesions with pulpal involvement were excluded from the study. The selected teeth were then randomly assigned into three experimental groups (n=7), each representing a different caries removal methodology: Group 1 (SmartPrep bur), Group 2 (chemomechanical removal), and Group 3 (air abrasion). Following group allocation, all specimens were immersed in distilled water and stored at a controlled temperature of 4°C until their subsequent utilization.

- Sample Preparation

**Figure 1:**
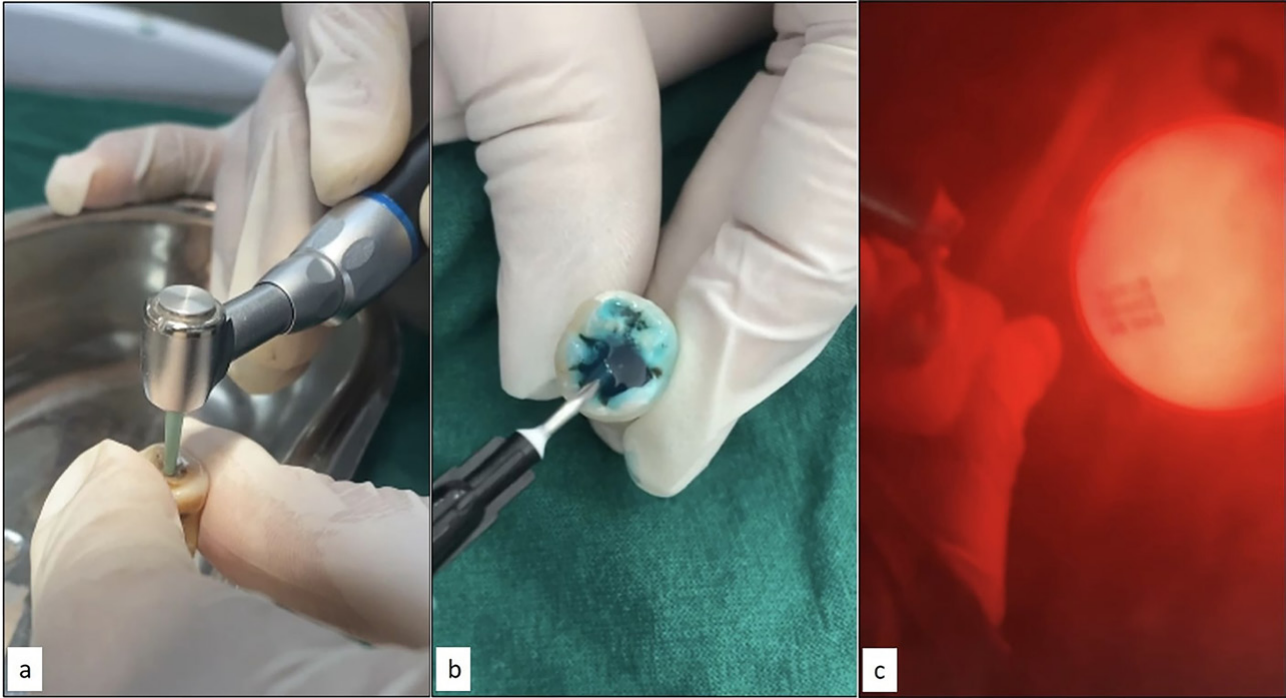
(a) Caries excavation using polymer bur, (b) Papacarie gel application, (c) caries excavation using air abrasion.

The efficacy of caries removal was assessed based on standardized visual and tactile criteria as established by Ericson *et al*. In Group 1 (SmartPrep Bur Protocol), caries excavation was carried out using calibrated circular movements, beginning at the central aspect of the lesion and advancing peripherally. The excavation process was discontinued upon observation of visible abrasion and the mechanical inefficacy of the SmartPrep bur (SmartPrep, SS White Burs, Inc., Lakewood, NJ, USA). For Group 2 (Chemomechanical Protocol), carious dentin was subjected to treatment with Papacarie® gel (Formula & Acao, Sao Paulo, Brazil) for a duration of 40 seconds. The softened carious tissue was then meticulously excavated using a Hu-Friedy 18 Excavator (EXC18, USA). The gel was replenished as needed upon the appearance of turbidity, and the process continued until the gel maintained clarity, indicating the thorough removal of demineralized tissue. In Group 3 (Air Abrasion Protocol), cavity preparation was achieved using 27 μm aluminum oxide particles propelled at an air pressure of 201 psi for 60 seconds. A standardized working distance of 5 mm and an angulation of 90° to the dentin surface were maintained throughout the procedure. Following air abrasion, specimens were subjected to a 20-second irrigation process to remove residual particles. To ensure procedural consistency and eliminate inter-operator variability, all caries removal procedures were conducted by a single experienced operator who had undergone rigorous training and calibration, (Fig. 1).

- Assessment & Evaluation

After the caries removal procedures, the specimens underwent horizontal cross-sectioning at the occlusal third, approximately 1 mm above the prepared surface, using a water-cooled diamond disk (Isomet 1000, Buehler Ltd., Lake Bluff, IL, USA). The sectioned samples were then preserved in a 0.1% thymol solution in deionized water before proceeding with adhesive application. Adhesive procedures followed the manufacturer’s specifications for 3M™ ESPE™ Adper™ Single Bond 2. A standardized plastic cylinder, with an internal diameter of 5 mm and a height of 5 mm, was carefully positioned on the dentin surface. The adhesive was photopolymerized using the Dentsply SmartLite Focus Curing Light for 15 seconds. The cylinder was subsequently filled with resin composite (Beautifil Bulk Flowable, Shofu, Kyoto, Japan) and underwent photopolymerization for an additional 40 seconds. Upon completion of polymerization, the plastic cylinder was removed using a #11 scalpel blade, leaving behind a perpendicular composite rod with precise dimensions (diameter: 5 mm, height: 5 mm). Dimensional accuracy was verified using digital vernier calipers (Mitutoyo, Kawasaki, Kanagawa, Japan). The specimens were then vertically sectioned along the buccolingual plane using a low-speed diamond disk, resulting in two symmetrical halves. The selected sections were subsequently embedded in self-curing acrylic resin (DPI RR Cold Cure Acrylic) to facilitate further analysis. To ensure adequate hydration before testing, all specimens were stored in distilled water at 37°C for a period of 24 hours.

- Statistical Analysis

The microtensile bond strength (μTBS) of the specimens was evaluated using a Universal Testing Machine (4411, Instron Co., Canton, MA, USA). The values for microtensile bond strength were expressed in megapascals (MPa) and calculated using the formula: MPa = Applied force at failure (N) / Bonded surface area (mm²). Comparative statistical analyses were performed to assess differences between groups. A one-way ANOVA test was employed to analyze continuously distributed data across the groups, while Tukey’s post hoc test was used for pairwise comparisons. A significance level of *p* < 0.05 was established as the criterion for statistical relevance in all analyses.

## Results

The comparative analysis of three caries removal techniques revealed distinct patterns in their efficacy and reliability. SmartPrep burs demonstrated superior performance with the highest mean tensile strength (33.31 ± 10.23 MPa; 95% CI: 22.57–44.04), although this was accompanied by considerable variability in the measurements. Chemo-mechanical removal techniques yielded intermediate results (22.86 ± 11.61 MPa; 95% CI: 10.67–35.04), while air abrasion exhibited the lowest mean tensile strength (15.74 ± 2.99 MPa) but demonstrated the most consistent performance as evidenced by its narrow confidence interval (95% CI: 12.60–18.89), ( Table 1).

Statistical evaluation using one-way ANOVA revealed significant differences among the groups. (F(2,15) = 5.652, *p* = 0.015), with the between-groups sum of squares (936.523, df = 2) exceeding the within-groups sum of squares (1242.809, df = 15), indicating meaningful variation among the techniques ( Table 2). Subsequent post hoc analysis using Tukey’s HSD test revealed that the most statistically significant difference in tensile strength existed specifically between the SmartPrep burs and air abrasion groups (*p* = 0.012) ( Table 3). These findings suggest that while SmartPrep burs may offer superior tensile strength, the choice of technique may need to balance this advantage against the more consistent, albeit lower, performance of air abrasion, depending on the specific clinical requirements.

## Discussion

Contemporary minimally invasive dentistry fundamentally emphasizes restricted endodontic intervention in the management of dentin caries lesion (15). The paradigm shift toward conservative and preventive methodologies has transformed contemporary dental caries management (8). In response to the limitations of conventional rotary instrumentation, numerous alternative techniques have emerged, including chemo mechanical methods, Caries indicating dyes, laser applications, ultrasonic equipment, air abrasion,sono-abrasion, and polyamide bur (16). The recognition that caries-affected dentin serves as the primary substrate for restorative material interaction, coupled with demonstrably reduced adhesive strength compared to sound dentin, emphasizes the critical need for enhanced substrate adhesion (9).

The present *in vitro* investigation quantitatively assessed microtensile bond strengths of a two-step, etch-and-rinse adhesive system to caries-affected dentine following application of three distinct minimally invasive techniques for caries removal. The initial null hypothesis was contradicted by the experimental data, demonstrating statistically significant variations in bonding values among smartprep bur, air abrasion, and chemo-mechanical removal methodologies. Surface morphological variations resulting from different caries removal techniques exhibited significant influence on bond strength outcomes (1). A notable methodological limitation in current literature persists: *in vitro* bond strength studies predominantly employ flat, non-carious dentine substrates, potentially compromising clinical relevance (1).

Quantitative analysis demonstrated that selective caries removal utilizing the Smart Prep Bur achieved superior bond strength values, followed by the chemo-mechanical protocol, while air abrasion demonstrated significantly reduced values. These findings correlate with Mohan I *et al*.’s observations regarding SmartPrep burs’ enhanced capacity for debris elimination and smear layer removal, facilitating patent dentinal tubules and optimized adhesive restoration receptiveness (15). The SmartPrep bur, fabricated from biocompatible polymer with mechanical properties precisely calibrated to be marginally inferior to sound dentin, exhibits diminished cutting efficacy upon contact with sound or carious dentin, generating vibrational feedback that enables selective removal of demineralized tissue while preserving underlying dentin structure (4,16).

Chemomechanical caries excavation methodology employs a solution-based protocol targeting the surface infected, non-remineralizable layer of carious dentin (2). Chemomechanical caries removal gel-based formulation incorporates purified PAPAIN enzyme, extracted from Carica Papaya, supplemented with clove oil and chloramine (17). This pepsin-homologous enzyme demonstrates dual bactericidal and bacteriostatic properties. Flindt *et al*. elucidated papain’s selective action mechanism, which specifically targets infected dentin, attribuTable to the absence of A1-antitrypsin, thereby enabling selective degradation of compromised molecular structures while maintaining sound dentin integrity (17,18).

Air abrasion technology, while theoretically aligned with minimal intervention principles, generates surface roughness potentially conducive to bonding material retention (14). Implementing Sano *et al*.’s micro-tensile bond test methodology, this investigation utilized a standardized 2 mm² surface area for evaluation (19). The observed reduction in bond strength in the air abrasion group may be attributed to irregular surface morphology and dentinal tubule obstruction resulting from 27 μm aluminum oxide particle impingement (14).

The diminished bond strength observed in the chemo-mechanical group compared to the Smart Bur group may be attributed to residual caries-infected smear layers and tubule obstruction (1). Additional factors potentially contributing to reduced bonding values include residual papain gel retention and dentinal fluid exudation (1). Morphological analysis revealed that while both chemo-mechanical and Smart Bur groups demonstrated comparable resin tag formation, the Smart Bur group produced optimized surface characteristics, characterized by uniform surface topology with characteristic smear layer and patent dentinal tubules (16). The predominance of adhesive failure modes across all experimental groups aligns with Sardella *et al*.’s findings, while concurrent investigations validate polymer bur technology as a clinically viable alternative to conventional techniques, offering effective caries removal with enhanced patient comfort without necessitating local anesthesia (21).

The experimental design inherent to *in vitro* investigations presents methodological constraints in replicating the complex oral environment. The absence of critical physiological parameters—including salivary dynamics, fluctuating pH gradients, and cyclic masticatory forces—may limit the direct clinical extrapolation of these findings. Moreover, the intrinsic heterogeneity of caries-affected dentin, characterized by variations in structural integrity, mineral density distribution, and lesion progression depth, introduces potential confounding variables in adhesive interface evaluation. The temporal limitations of this investigation preclude assessment of long-term bond stability. Degradation mechanisms, including hydrolytic breakdown of the adhesive interface, matrix metalloproteinase-mediated collagen degradation, and thermomechanical fatigue from repeated thermal and mechanical cycling, remain unexamined. These factors could significantly influence the longevity and clinical performance of the adhesive interface over extended periods. Future investigations incorporating artificial aging protocols, dynamic loading conditions, and comprehensive analysis of the adhesive interface’s physicochemical properties would enhance our understanding of these bonding systems’ long-term clinical efficacy. Additionally, in situ studies examining the impact of the oral microenvironment on bond integrity could provide more clinically relevant insights.

The experimental findings demonstrate a statistically significant correlation between caries removal methodology and resultant bond strength values to caries-affected dentin. Quantitative analysis revealed hierarchical bond strength values, with the polymer-based SmartPrep bur exhibiting superior microtensile bond strength, followed by chemomechanical intervention, while air abrasion demonstrated comparatively reduced adhesive efficacy. These results underscore the critical influence of surface micromorphology and patent dentinal tubule exposure on adhesive interface formation and stability. The differential bonding outcomes observed across treatment modalities have substantial implications for clinical protocol optimization. The superior performance of the polymer bur system suggests that the resultant surface characteristics may provide more favorable conditions for adhesive infiltration and hybrid layer formation. These findings provide an evidence-based framework for clinical decision-making in restorative dentistry, particularly regarding the selection of caries removal methodology. Future investigations should incorporate variables that more accurately simulate the complex oral environment, including the use of naturally carious substrates with varying degrees of demineralization, and the implementation of mechanical and thermal cycling protocols. Such methodological refinements would enhance the clinical relevance and predictive value of *in vitro* bonding studies, ultimately contributing to more effective therapeutic strategies in adhesive dentistry.

## Figures and Tables

**Table 1:** Comparison of mean microtensile bond strength between the three groups.

	N	Mean	Std. Deviation	95% Confidence Interval for Mean		Minimum	Maximum
				Lower Bound	Upper Bound		
Smart bur	7	33.3067	10.23203	22.5688	44.0445	24.70	48.70
Air abrasion	7	15.7433	2.99433	12.6010	18.8857	11.76	19.68
Chemo Mechanical	7	22.8583	11.61470	10.6695	35.0472	11.22	44.38

**Table 2:** One-way variance of analysis comparing three groups.

	Sum of Squares	df	Mean Square	F	Sig.
Between Groups	936.523	2	468.262	5.652	.015
Within Groups	1242.809	15	82.854		
Total	2179.332	17			

**Table 3:** Post hoc analysis with Tukey’s test for multiple comparisons.

Multiple group evaluations						
(I) group	(J) group	Mean Difference (I-J)	Std. Error	Sig.	95% Confidence Interval	
					Lower Bound	Upper Bound
Smart bur	Air abrasion	17.56333^*^	5.25528	.012	3.9129	31.2138
	Chemo Mechanical	10.44833	5.25528	.149	-3.2021	24.0988
Air abrasion	Smart bur	-17.56333^*^	5.25528	.012	-31.2138	-3.9129
	Chemo Mechanical	-7.11500	5.25528	.389	-20.7654	6.5354
Chemo Mechanical	Smart bur	-10.44833	5.25528	.149	-24.0988	3.2021
	Air abrasion	7.11500	5.25528	.389	-6.5354	20.7654

*. The mean difference is significant at the 0.05 level.

## Data Availability

The datasets analysed during the present investigation are available from the corresponding author on request.
